# A Case Study: Electrically Assisted Stress Relief Annealing for Cold-Coiled Helical Automotive Springs

**DOI:** 10.3390/ma17081774

**Published:** 2024-04-12

**Authors:** Van Loi Tran, Sung-Tae Hong, Ji Ye Hong, Tae Shik Yeo

**Affiliations:** 1 School of Mechanical Engineering, University of Ulsan, Ulsan 44610, Republic of Korea; tranvanloi.bg90@gmail.com; 2 Research & Development Center, Daewon Kang Up Co., Ltd., Cheonan-si 31042, Republic of Korea; hongjy@dwku.com (J.Y.H.); yeotaeshik@dwku.com (T.S.Y.)

**Keywords:** electrically assisted, residual stress, stress relief annealing, cold-coiled automotive springs

## Abstract

This study experimentally investigated electrically assisted (EA) stress relief annealing for cold-coiled commercial automotive springs. In EA stress relief annealing, the temperature of a spring is rapidly increased to the annealing temperature (400 °C) and is held constant for a specified time using a pulsed electric current. Experimental findings show that the effectiveness of the EA stress relief annealing is superior to that of the conventional stress relief annealing, especially in terms of process time. The present study suggests that EA stress relief annealing, with properly selected process parameters, can effectively substitute for time-consuming conventional stress relief annealing using a furnace for cold-coiled automotive springs.

## 1. Introduction

Helical springs manufactured by cold coiling have been widely used for automotive suspensions. However, cold coiling causes non-uniform residual stress distribution in the spring, which can significantly reduce product life and cause dimensional variation [1]. Therefore, in the commercial manufacturing process of helical automotive springs, residual stress associated with cold coiling needs to be appropriately reduced or eliminated by stress relief annealing to meet the durability requirement. Conventional stress relief annealing is generally carried out at 400 °C for 40 min in a furnace to reduce residual stresses and eliminate weak spots. However, conventional stress relief annealing is time-consuming, requires bulky equipment, and increases production costs. Therefore, the automotive spring industry would welcome a faster and more compact alternative.

Electrically assisted manufacturing (EAM) utilizes the combined effect of resistance heating and the athermal effect of electric currents [2,3,4] to control the mechanical behavior and microstructure of metal alloys. This relatively new approach has been actively researched and evaluated for various practical applications, such as bending [5], blanking [6], drawing [7,8], forging [9,10], springback reduction [11], and rolling [12]. With the ability to improve efficiency, accuracy, and material performance, EAM has the potential to replace various conventional manufacturing processes.

EA stress relief annealing is an EAM technique that uses the thermal and athermal effects of electric current to improve the efficiency of the annealing process. Nguyen et al. [13] evaluated the athermal contribution of electric current during EA annealing by comparing the performance of EA annealing and rapid induction heat annealing. In the research of Park et al. [14], a semicircular specimen prepared from a cold-coiled automotive spring was used to confirm the feasibility of EA stress relief annealing. Their experimental results showed that the effect of EA stress relief annealing could be similar to that of conventional stress relief annealing using a furnace, even with a significantly shorter process time and lower energy.

As a follow-up to the research of Park et al. [14], in the current study we expanded the EA stress relief annealing concept to full-size commercial automotive springs manufactured by cold coiling. The effect of EA stress relief annealing parameters on the residual stress distribution in the full-size spring was evaluated. Additionally, the resulting mechanical properties of the full-size spring after EA stress relief annealing were compared with those after conventional stress relief annealing using a furnace.

## 2. Experimental Set-Up

### 2.1. Materials

**Figure 1:**
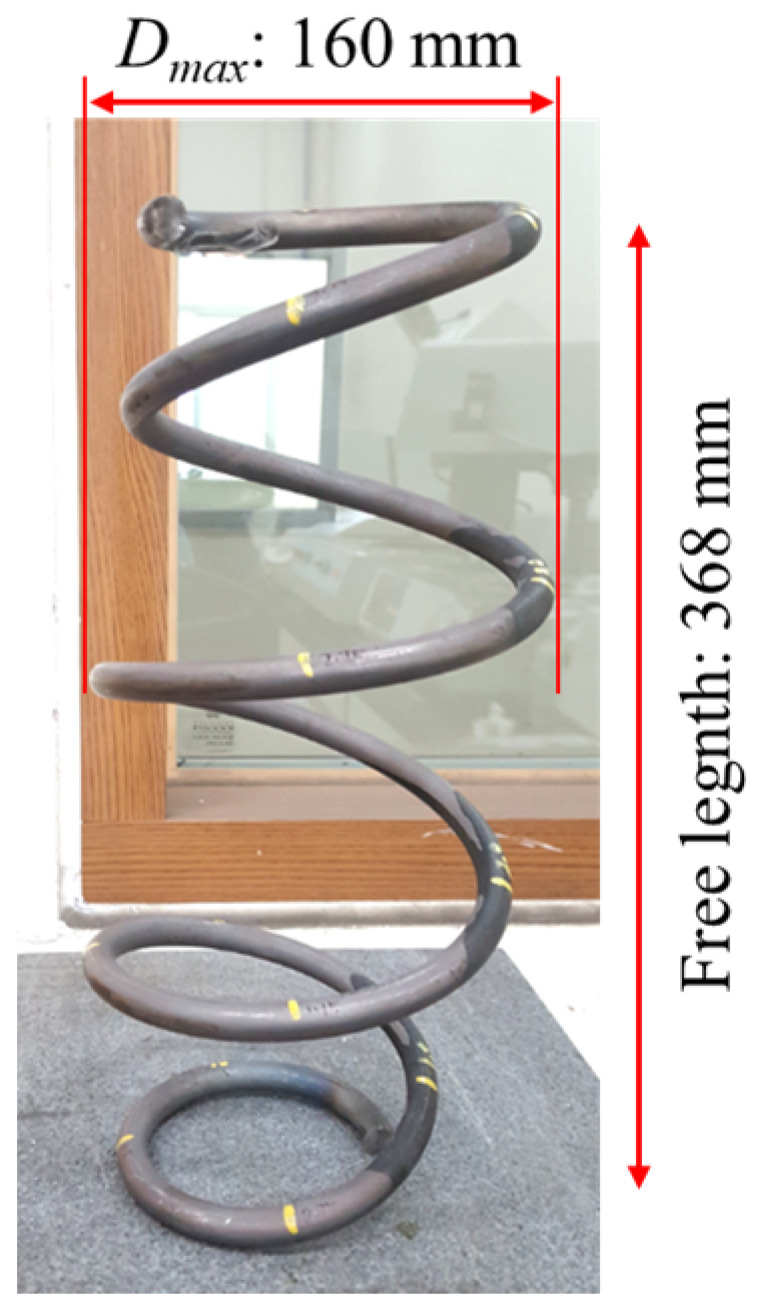
Geometry and dimensions of spring specimens.

The cold-coiled springs used for the stress relief annealing experiment were manufactured from high-strength steel round bars (Si-Cr-V spring steel, supplied by POSCO, Pohang-si, Republic of Korea) with a diameter of 13.0 mm (chemical composition: property of the manufacturer). The steel bar was quenched and tempered before being formed into the shape of the spring [15,16]. All the spring specimens were selected from the same production run to ensure consistency in the experimental results. The height of each spring specimen was 368 mm with 4.75 coils, while the upper and lower outer diameters were slightly smaller than the middle outer diameter, as shown in Figure 1.

### 2.2. Effectiveness of EA Stress Relief Annealing

**Figure 2:**
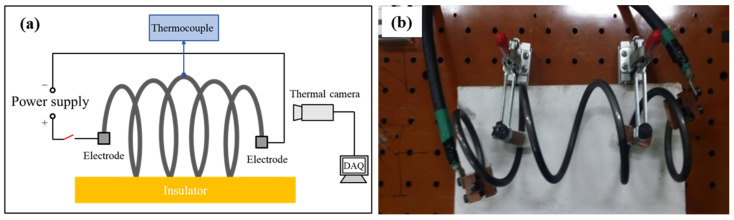
(**a**) Schematic of experimental set-up; (**b**) photograph of experimental set-up.

**Figure 3:**
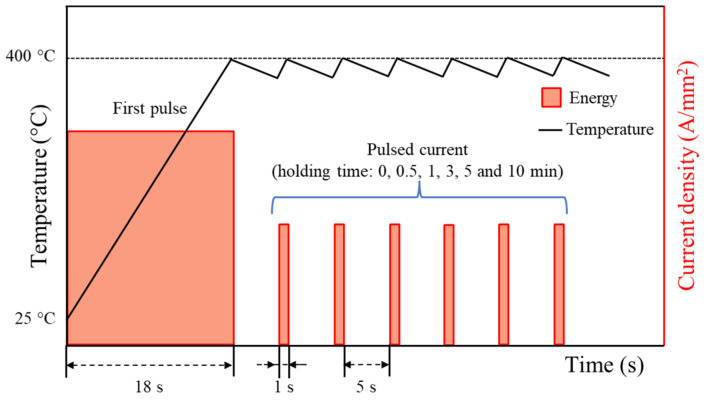
Schematic of the experimental parameters for EA stress relief annealing.

For the EA stress relief annealing experiment, a full-size spring specimen was mounted on an insulating baseplate, and electric current was applied from one tip of the spring to the other, as described in Figure 2. The electric current was generated by a programmable electric current generator (Vadal SP-1000U, Hyosung, Seoul, Republic of Korea). In the EA stress relief annealing experiment, the cold-coiled spring was rapidly heated to the annealing temperature of 400 °C by an initial electric current for 18 s. Then, the specimen was held at the annealing temperature for a specific holding time by applying a pulsed electric current with a relatively lower amplitude, as schematically described in Figure 3 and listed in Table 1. As shown in Table 1, the electric current density was defined as the amount of electric current flowing through a unit cross-sectional area of the spring wire. After EA stress relief annealing, the spring specimen was cooled to room temperature in air. For each experimental condition listed in Table 1, at least three specimens were tested to confirm the repeatability of the result. The temperature history of the specimen during the test was measured using an infrared thermal imaging camera (T440, FLIR, Täby, Sweden). The emissivity of the thermal images was calibrated using the temperature measured using a K-type thermocouple and a data logger (MV-106, Yokogawa, Musashino-shi, Japan) at the location of 2.5 coils, which was the center of the spring specimen.

To evaluate the effectiveness of EA stress relief annealing, a conventional stress relief annealing experiment was carried out in which a spring was annealed in a laboratory furnace using a commercial heat treatment schedule provided by the spring manufacturer. The temperature reached the annealing temperature (400 °C) after about 20 min and was held constant for an additional 20 min. Then, the sample was removed from the furnace and cooled in air. Therefore, the total process time for the conventional stress relief annealing experiment was approximately 40 min. During conventional stress relief annealing, the specimen temperature was measured using a K-type thermocouple at the center of the spring specimen.

The performance of stress relief annealing was evaluated by measuring the residual stress distribution in the spring after annealing. An X-ray diffractometer (MSF/PSF-3M, Rigaku, Tokyo, Japan) was used to measure residual stress distribution along the length of the spring at the inner surface of as-coiled, EA stress relief-annealed, and conventionally annealed springs. Thereafter, the mechanical property of the spring specimen was evaluated by measuring the Vickers hardness profile (HM-100, Mitutoyo, Kawasaki city, Japan) along the diameter of the cross-section at the center of the spring specimen (2.5-coil location) with a measurement distance of 0.25 mm and a load of 9.8 N.

### 2.3. Effect of Energy Release Rate

**Figure 4:**
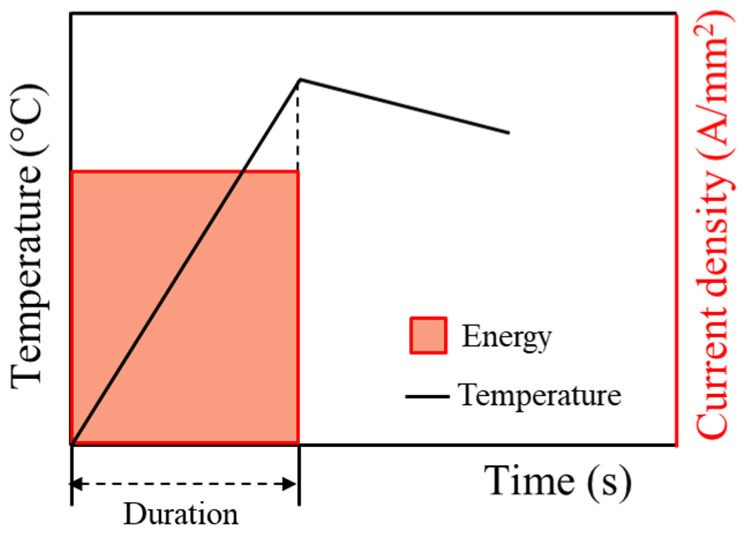
Schematic of the experimental parameters for energy rate test.

The effect of electric energy rate, which was defined by the energy input divided by the duration of initial electric current during the heating to the annealing temperature of 400 °C, was evaluated by a series of EA stress relief annealing experiments with the same total energy input but at different electric current densities and durations. The effect of electric energy rate was evaluated in a single-pulse mode, as schematically described in Figure 4. The test parameters are listed in Table 2. No temperature hold at the annealing temperature was attempted for simplicity of the study. Once the specimen temperature reached the annealing temperature, the specimen was cooled to room temperature in air. The total energy input by electric current was calculated using the following formula:

(1) $$E=\Sigma I^{2}R∆t=R.\Sigma I^{2}t=\rho \frac{L}{A}\Sigma I^{2}∆t$$

where *E* is total electric energy (J), *I* is electric current (A), *R* is resistance (Ω), and Δ*t* is the duration of electric current (s). The total energy *E* for the energy rate test was selected to match the electric energy input during the initial heating of the above experiment for effectiveness of EA stress relief annealing.

## 3. Results and Discussion

### 3.1. Effectiveness of EA Stress Relief Annealing

**Figure 5:**
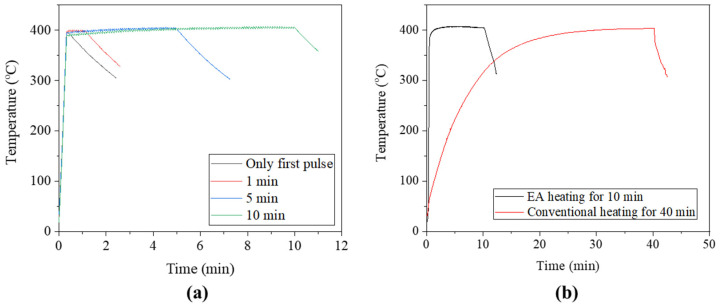
(**a**) Temperature profiles of EA stress relief annealing (0, 1, 5, and 10 min processing times); (**b**) compared with conventional heating in the furnace (40 min process times).

The specimen temperature rapidly increased to the annealing temperature and was held nearly constant during the remaining process time, as shown in Figure 5a. In comparison, the conventional annealing approach required approximately 20 min to reach the annealing temperature prior to an additional 20 min at the annealing temperature (Figure 5b). A major advantage of EA stress relief annealing over conventional annealing is the ability to reach the target temperature very rapidly due to the Joule heating effect of the current.

**Figure 6:**
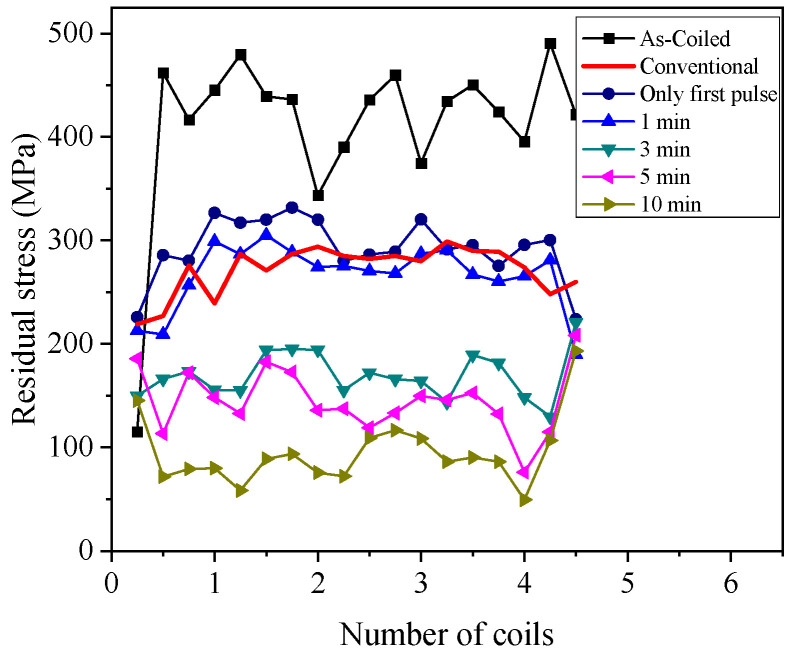
The results of residual stress along the inner surface of as-coiled, EA-annealed, and conventionally annealed springs.

The residual stress along the length at the inner surface of the as-coiled spring specimen is tensile stress, which adversely affects the durability of the spring by reducing its fatigue life. Additionally, the helical spring design in the present study has varying outer diameters and pitches along the length. Thus, the as-coiled spring had a significantly varying inner residual stress distribution along the length, as shown in Figure 6. As a result of stress relief annealing, the residual stress distribution of the spring specimen became much more even (less fluctuation along the length of the spring), regardless of the annealing method. In addition, the residual stress decreased as the holding time of EA stress relief annealing increased. Most importantly, in EA stress relief annealing, even with a holding time of less than 1 min, the residual stress reached a distribution similar to that of conventional annealing for 40 min. This clearly confirms the superior efficiency of EA stress relief annealing over the conventional method using a furnace. The superiority of EA stress relief annealing can be explained by the combined effect of elevated temperature by resistance heating and the athermal effect of electric current, which additionally enhances the mobility of metal atoms [17].

**Figure 7:**
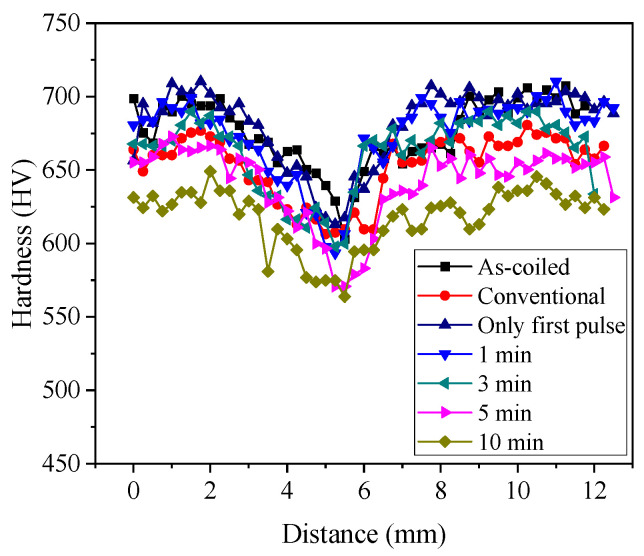
The micro-hardness results of as-coiled, EA-annealed, and conventionally annealed springs.

The hardness distribution of EA stress relief annealing specimens also decreased as holding time increased (Figure 7). Naturally, the hardness on the outside of the cross-section was larger than that at the center due to the plastic deformation from cold coiling. The effect of EA stress relief annealing on the micro-hardness of spring specimen was also more substantial than that of conventional annealing. This result suggests that even a holding time of 10 min was excessive for EA stress relief annealing. The experimental results of the present study showed that EA stress relief annealing was feasible with a substantially shorter process time and considerably lower energy than conventional furnace-based processes.

### 3.2. Effect of Energy Release Rate

**Figure 8:**
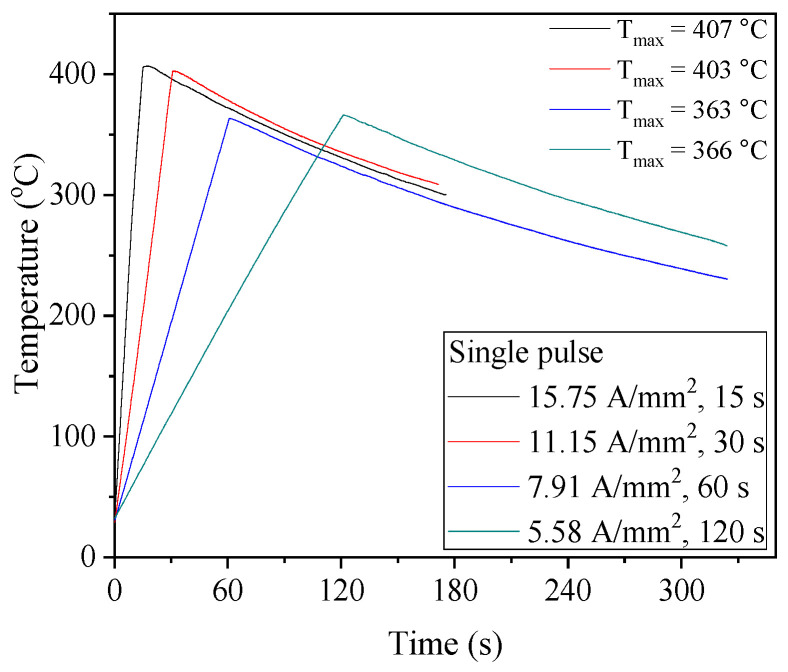
Temperature profiles of energy release rate test.

In the energy release rate experiment, the peak temperature decreased with an increase in the duration of current density due to the heat transfer to the ambient air despite the same energy input, as shown in Figure 8. The peak temperatures were in the range of 363 to 407 °C below the recrystallization temperature for the spring steel. After the peak temperature, the specimens were cooled in air to room temperature, and the cooling rates were similar.

**Figure 9:**
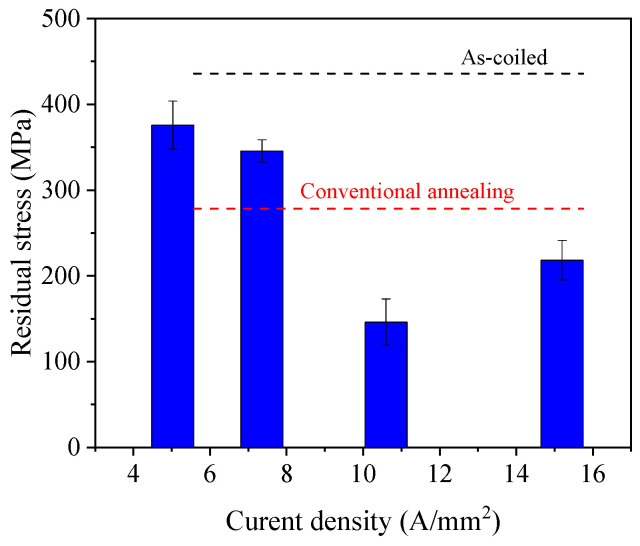
The results of residual stress at 2.5-coil location with different energy rates.

The residual stress measurement results show that the energy release rate significantly affects the spring residual stress, as shown in Figure 9. Based on the comparison between the results with similar peak temperatures (407 and 403 °C), the results indicate that the efficiency of EA stress relief annealing was lower with higher electric current density combined with shorter duration to apply the same electric energy. This result suggests that for the process parameter range selected in the present study, among the two main factors deciding the efficiency of the EA stress relief annealing, the effect of elevated temperature decreased more significantly due to the shorter heating time (shorter exposure of the specimen to the elevated temperature) than the effect of increased electric current density [18]. Consequently, these results strongly suggest that the EA stress relief annealing parameters (current density and duration) should be properly combined to optimize the performance of EA stress relief annealing.

### 3.3. Validation of the Effectiveness of EA Stress Relief Annealing

**Figure 10:**
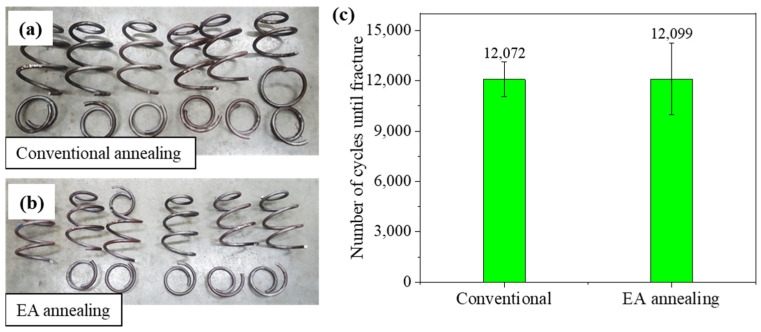
Comparison of fatigue life between conventional and EA stress relief annealing: (**a**) conventional specimens, (**b**) EA-annealed specimens, (**c**) number of cycles until fracture.

**Figure 11:**
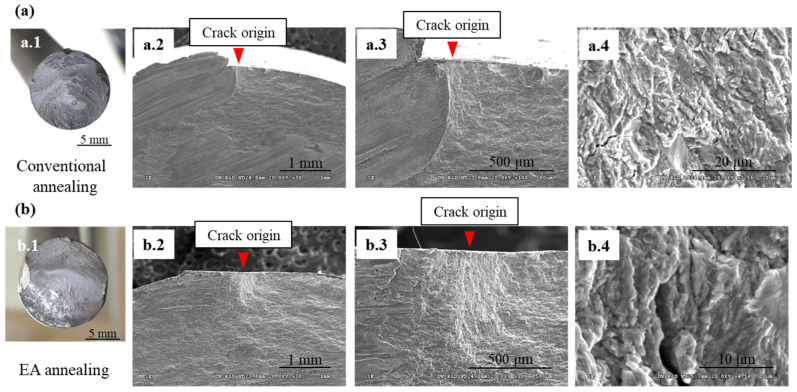
The crack origin zones of the fracture surfaces of two different annealing methods: (**a**) conventional and (**b**) EA annealing; (**a.1**,**b.1**) photographs of the fracture surface, (**a.2**,**b.2**) SEM photographs of the fatigue origin, (**a.3**,**b.3**) close view of the crack origin, (**a.4**,**b.4**) magnified views of the crack origin.

The EA stress relief-annealed spring with a holding time of 30 s was employed for the fatigue test (without shot peening). The fatigue life of the EA-annealed spring was similar to that of a conventionally annealed spring (~12,000 cycles) under the same loading condition (stress amplitude from 200 to 1200 MPa) (Figure 10). Most spring specimens were fractured at the 1.5-coil position, as shown in Figure 10a,b. Optical microscopy of fracture surfaces showed that the crack originated from the inner surface and propagated over the spring cross-section for both cases (Figure 11). The initial fatigue crack can grow in areas of high tensile residual stress. Previous studies have shown that tensile residual stress distributed on the inner surface of the spring tends to accelerate the initiation and progression stages of the fatigue process [15,19]. Micro-cracks were observed on the fracture surfaces at high magnification, which is characteristic of the brittle fracture mechanism (Figure 11(a.4,b.4)).

The similar fatigue life of the EA-annealed spring to that of the conventionally annealed spring suggests that an additional process, typically shot peening (used to introduce compressive residual stress on the surface of the spring), will be still necessary even with EA stress relief annealing. However, the superiority of EA stress relief annealing over conventional annealing with a furnace is still valid and impressive, since the approximately 30 s long EA stress relief annealing resulted in a fatigue life comparable to the 40 min long conventional annealing.

### 3.4. Combined EA Rapid Heating and Hot Setting

**Figure 12:**
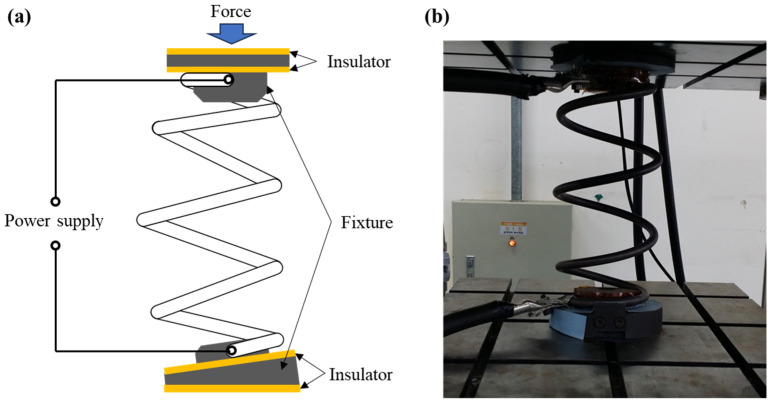
(**a**) Schematic; (**b**) photograph of the experimental set-up for hot setting.

EA stress relief annealing can also be combined with a hot setting in automotive spring production. The hot setting (plasticization) allows the spring to achieve the correct free length. Conventionally, the stress relief-annealed spring was heated again in the furnace, moved to the compressor, and quickly compressed at a temperature of 330 °C. This process is time-consuming and requires a lot of accompanying equipment. In the EA hot setting, a custom-made fixture was installed in a programable press to fix the specimen at both ends, as illustrated in Figure 12. A hot setting process to adjust the free length of the spring was successfully conducted as a part of the EA stress relief annealing.

**Figure 13:**
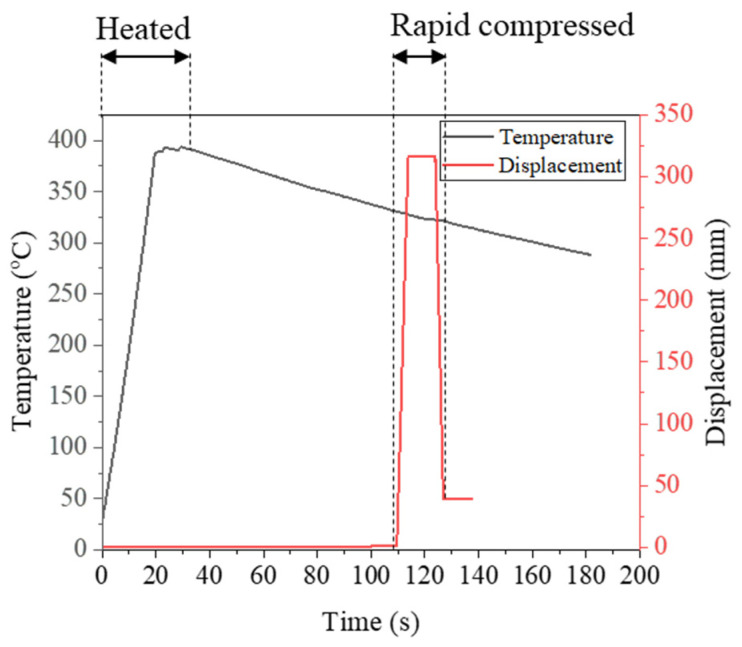
Temperature profiles and displacement in hot setting test.

**Figure 14:**
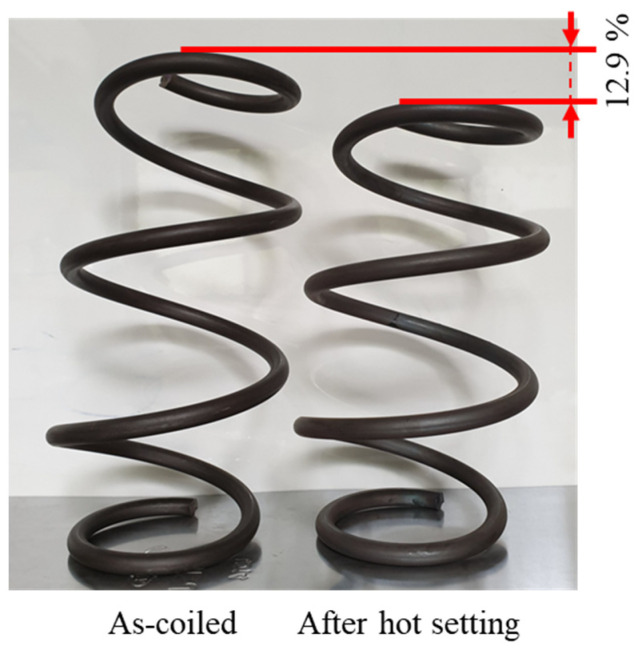
Spring specimens before and after hot setting test.

As shown in Figure 13, the spring specimen was EA stress relief annealed at about 400 °C with a holding time of 30 s. Then, during air cooling after annealing, the spring specimen was fully compressed at a speed of 30 mm/s at a temperature of 330 °C, a typical hot setting condition. After compression, the specimen was kept in the fully compressed position for 10 s and then released at the same speed. As a result, the free height of the spring specimen successfully reached the target value of hot setting (Figure 14). By combining the EA stress relief annealing and EA hot setting in a single step, the manufacturing process of the cold-coiled automotive spring can be further simplified with a shorter cycle time.

## 4. Conclusions

The present study demonstrated the EA stress relief annealing concept for cold-coiled helical automotive springs. The effectiveness of the proposed EA process was assessed through a comparison with the outcomes of conventional stress relief annealing. The experimental findings unequivocally indicate that EA stress relief annealing can be superior to a conventional stress relief process with notable reductions in processing time and energy consumption. Furthermore, the fatigue analysis results confirmed that the EA stress relief annealing satisfied the industrial specifications for commercial automotive springs.

## Figures and Tables

**Table 1:** EA stress relief annealing parameters.

First Pulse (Heating)		Repeated Pulse (Holding)			Holding Time (min)
Current Density (A/mm^2^)	Duration (s)	Current Density (A/mm^2^)	Duration (s)	Period (s)	
13.75	18	7.15	1	5	0, 1, 3, 5, 10

**Table 2:** EA stress relief annealing parameters for energy rate test.

Number of Pulses	Current Density (A/mm^2^)	Duration (s)	Total Energy (J/Ω)
1	15.75	15	65.52
1	11.15	30	65.71
1	7.91	60	66.15
1	5.58	120	65.71

## Data Availability

The raw/processed data required to reproduce these findings cannot be shared at this time as the data also form part of an ongoing study.
